# Environmentally Friendly Polymer Blends Based on Post-Consumer Glycol-Modified Poly(Ethylene Terephthalate) (PET-G) Foils and Poly(Ethylene 2,5-Furanoate) (PEF): Preparation and Characterization

**DOI:** 10.3390/ma13122673

**Published:** 2020-06-12

**Authors:** Sandra Paszkiewicz, Izabela Irska, Elzbieta Piesowicz

**Affiliations:** Department of Materials Technologies, West Pomeranian University of Technology in Szczecin, Piastow 19 Av., PL-70310 Szczecin, Poland; iirska@zut.edu.pl (I.I.); elzbieta.piesowicz@zut.edu.pl (E.P.)

**Keywords:** polymer blends, poly(ethylene 2,5-furanoate), post-consumer PET-G foils

## Abstract

Environmentally friendly polymer blends between post-consumer PET-G and bio-based poly(ethylene 2,5 furanoate) (PEF) have been prepared. The PET-G granules were obtained from the post-consumer glycol-modified poly(ethylene terephthalate) PET-G foils from Nicrometal S.A. as a result of materials recycling. PEF was synthesized from dimethyl furan-2,5-dicarboxylate and 1,2-ethylene glycol (BioUltra) by a two-stage melt polycondensation process. According to the calculations followed by Hoy’s method, one has studied the miscibility of the components in the blend. The molecular structure of PET-G/PEF blends was analyzed by Fourier Transform Infrared Spectroscopy (FTIR) spectroscopy, while the morphology of the blends was determined by Scanning Electron Microscopy (SEM). To evaluate phase transition temperatures, as well as the thermal effects in PET-G/PEF blends, Differential Scanning Calorimetry (DSC), Dynamic Mechanical Thermal Analysis (DMTA), and Thermogravimetric Analysis (TGA), were performed. Tensile tests revealed that along with an increase in the amount of PEF, an increase in Young’s modulus was observed. Besides, the existence of interfacial interactions between polymers, especially in the case of PET-G/PEF 80/20, enabling the PET-G chains to form a network structure with the PEF by reacting with their functional groups, allows observation of a synergistic effect in the improvement of thermal stability and water absorption.

## 1. Introduction

Polymer blending is an efficient and inexpensive method of diversification of polymer properties [1,2,3]. To obtain a useful product that can be classified as miscible or mechanically compatible [2,4], i.e., with a certain level of cohesion, one should consider the various structural parameters of the components that constitute the blend. Recently, the most important challenge faced by polymer blending technology is the ability to use recycled polymers for the preparation of blends, thereby preventing the use of natural resources and reducing environmental pollution [3]. It is said that if we do not stop polluting the oceans with plastic, in 2050, more plastic bottles will float in them than fish. Plastic raises a lot of controversies. Not only environmentalists are protesting against its use [5]. It is particularly dangerous to use it for the mass production of packaging, disposable dishes, perishables, which are mainly obtained from thermoplastic polyesters (poly(ethylene terephthalate) (PET), cycloaliphatic glycol-modified poly(ethylene terephthalate) (PET-G)) and polyolefins (polyethylene (PE), polypropylene (PP)). With a high probability, petrochemical plastics known to us today will be soon replaced with materials that will be biodegradable without harming the environment. However, by then, it is important to utilize existing post-consumer materials, for example, by putting them into circulation in another form, like in the form of the polymer blend. 

PET is an extensively used thermoplastic polyester due to its combination of unique physical, mechanical and barrier properties, and processability. As an engineering material, it offers, among others, excellent high-temperature properties, creep resistance, and solvent resistance [6]. Unlike PET, PET-G, depending on the amount of cycloaliphatic glycol [6], is an amorphous thermoplastic polyester with high transparency and clarity that exhibits a glass transition temperature (*T_g_*) of about 80 °C [7,8], which is excellent for extrusion, injection molding, blow molding and thermoforming [9]. The availability of large amounts of recycled polyesters, like as just mentioned PET or PET-G, largely from the packaging industry, electronics, and other applications [9,10,11,12,13,14], substantiates studies of their blends with other plastics. Nowadays, academic and industrial research in the field of polymer materials is strongly oriented towards not only the recycled polyesters but also to the bio-based alternatives to petroleum-derived plastics, with enhanced properties for advanced applications [12,15,16,17]. In this context, 2,5-furan dicarboxylic acid (FDCA) is a monomer readily accessible from sugars and is found to be one of the most potential bio-based building blocks for polymers [15,18,19] and is the first candidate to replace petrol-originated terephthalic acid. The polyesters, obtained from FDCA (or the corresponding derivatives, like dimethyl 2,5-furandicarboxylate (DMFDC)) with diols at different -CH_2_- amounts [20], have already exhibited interesting properties, mainly in the field of high barrier performances [15]. Moreover, its glass transition temperature is 88 °C [21], higher than that of PET and PET-G. In this regard, several studies are focused on PEF [22,23] as an attractive, viable bio-sourced substitute for PET, which is now dominating the packaging market, mainly in the soft drink bottle area [19]. Oxygen permeability in PEF is reduced by a factor of about 10 [21,22,24], and at the same time, carbon dioxide permeability is reduced by a factor of 19 [25,26] as compared to PET, which can be ascribed mainly to the reduction in chain flexibility in the presence of furan rings [22,27]. Thus, one can find furan-based polyesters to be good candidates for new excellent barrier materials. Besides, based on structural considerations, PET and its homologs like PET-G and PEF, are capable of specific interactions (e.g., H-bonding), but also, chemical reactions with a variety of other polar polymers. In this context, there are few studies on the preparation and characterization of PET-G blends with other polymers like PET [28,29], poly(butylene terephthalate) (PBT) [3,30], recycled PET-G [9], poly(ethylene naphthalate) (PEN) [31], polycarbonates (PC) [32], ethylene-vinyl copolymer (EVA) [33,34], PP [35], polyetherimide (PEI) [36] or even with ethylene propylene diene monomer (EPDM) [37]. For instance, in the work of Papadopoulou and Kalfoglou [29], the melt-mixed PET/PET-G blends show good mechanical properties at all compositions when quenched, and the annealing caused tensile properties reduction at high PET contents due to embrittlement. Besides, the important work of Latko et al. [9], where the mechanical recycling process of post-consumer PET-G foils was presented, which involved the removal of colored prints using an organic solution and subsequent extrusion the clear foil pieces to form regranulated pellets. In turn, there is also a certain number of studies on the PEF blends with different commercially important polymers [25,38,39]. Particularly noteworthy is the work of Papageorgiou et al. [25], who prepared PEF and PPF blends with PEN, PLA, PC, and PET. Based on these results, they assumed the potential of blending as a solution to face the main drawbacks of furan-based polymers which limit their industrialization and uses of the furanoate polyesters in industrial applications. Nevertheless, according to our knowledge, this is the first work on the novel environmentally friendly polymer blends based on two amorphous polyesters, i.e., post-consumer PET-G foil and bio-based PEF, with the comparable values of glass transition temperatures. The series of blends were studied concerning their miscibility, homogenization, mechanical performance, and thermal properties. Moreover, to face the possible limitations of such blends in industrialization, the water absorption tests were performed for the series of blends, neat PEF, and post-consumer PET-G foil.

## 2. Materials and Methods 

### 2.1. Preparation of Poly(Ethylene 2,5-Furanoate) (PEF)

The homopolymer of poly(ethylene 2,5-furanoate) (PEF) was synthesized by a two-stage melt polycondensation process. The synthesis setup consists of 1 dm^3^ steel reactor equipped with the condenser, stirrer, gas inlet, and a vacuum pump. The appropriate amount of the following substrates, i.e., DMFDC (99%, purchased from Henan Coreychem Co., Ltd., Henan, China), 1,2-ethylene glycol (ED, BioUltra, ≥99.5% (GC), purchased from Sigma-Aldrich) in a molar ratio of diester to diol 1:2, the first portion of catalyst (tetrabutyl orthotitanate, Ti(OBu)_4_, (Fluka)) and antioxidant (Irganox 1010 (Ciba—Geigy, Switzerland)) were inserted into the reactor and heated up to 165 °C. Since the beginning of the transesterification between DMFDC and ED (the byproduct, methanol, has started dripping), the reaction mixture was gradually heated up to the temperature of 200–215 °C. When the amount of the distilled methanol achieved 90% of the theoretical value (calculated stoichiometrically), the second portion of catalyst (also Ti(OBu)_4_,) was added, and the second stage, by means of melt polycondensation, began. The temperature in the reactor was gradually raised to 240 °C, while the pressure was reduced to ca. 25–30 Pa. Progress of this stage was monitored by an increase in the stirrer torque. Finally, the resulting homopolymer was extruded from the reactor under the nitrogen pressure, cooled to room temperature in a water bath, and then, granulated.

### 2.2. Preparation of PET-G pellets

**Figure 1:**
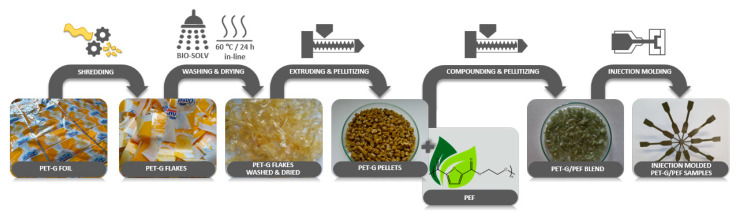
Schematic illustration of the preparation procedure of PET-G/PEF blends.

The PET-G granules were obtained from the post-consumer multilayer PET-G foils (obtained from Nicrometal S.A. as a result of materials recycling) that in the first step were subjected to grinding followed by removing the color printing (scheme presented in Figure 1). To remove the color print, one used Biosolv (Black Bear Boating and Leisure, England), which is a heavy-duty cleaner–biodegradable water-based degreaser (oil dispersant). The aim was to replace the acetone, the solvent that is normally utilized to remove the color prints from the foils, with the “bio-acetone”. Subsequently, the PET-G scraps were washed in water (to remove the solvent) and dried for 24 h under a dynamic vacuum at 60 °C. After drying, the PET-G flakes were fed into a counter-rotating twin-screw extruder (LSM30, Leistritz Laborextruder, Nuremberg, Germany) using the following parameters: feed zone: 20 °C; zone 1 and 2: 100 °C; zones 3–6: 180 °C; zones 7 and 8: 190 °C; the extruder screw speed was set at 60 rpm. In line, immediately after the extrusion, the granulation process was carried out.

### 2.3. Preparation of PET-G/PEF Blends

The series of PET-G/PEF reactive blends containing 20, 30, and 50 wt. % PEF were prepared by melt blending using a counter-rotating twin-screw extruder (LSM30, Leistritz Laborextruder, Nuremberg, Germany) with a 34 mm screw diameter and a length/diameter ratio of 23, equipped with two gravimetric feeders (Figure 1). In the compounding process, the following temperatures were determined: feed zone: 20 °C, zone 1: 100 °C, zone 2: 100 °C, zone 3: 170 °C, zone 4: 170 °C, zone 5: 170 °C, zone 6: 170 °C, zone 7: 180 °C, zone 8 (nozzle): 180 °C. For all blends, 40 rpm screw speed was used. The extruded materials were cooled in a water bath and granulated. Subsequently, dumbbell shape samples (A3 type) for DMTA and tensile testes were prepared according to the standard test methods ASTM D638-type V. The optimal injection pressure was around 60 MPa and the temperatures were ca. 15 °C higher than the softening temperatures of the blends determined by the Boetius method, since from the DSC, it was found that the neat polymers and blends were amorphous. An injection molding machine (Dr. Boy GmbH and Co., Neustadt-Fernthal, Germany) was utilized. The following injection parameters were applied: injection pressure 55 MPa, melt temperature (depending on the type of the material and set ca. 20 °C above the T_B_, so for PET-G: 150 °C; PET-G/PEF 80/20: 160 °C; PET-G/PEF 70/30: 175 °C; PET-G/PEF 50/50: 195 °C; PEF: 215 °C), mold temperature 30 °C, holding down pressure of 20 MPa for 15 s and cooling time of 10 s.

### 2.4. Characterization of PET-G/PEF Blends

The PET-G/PEF blends were characterized using an FTIR spectrophotometer (Bruker Optik GmbH model Tensor 27). Measurements were carried out using the attenuated total reflectance (ATR) technique. Each sample was scanned 32 times at the resolution of 2 cm^−1^ over the frequency range of 4000–400 cm^−1^.

The morphology of PET-G/PEF blends was characterized by scanning electron microscopy (SEM, Hitachi SU-70, Naka, Japan) with an accelerating voltage of 5kV and a working distance of 12 mm. The samples for SEM analysis were cryofractured in liquid nitrogen and subsequently coated (2–5 nm) in a vacuum with a thin gold film before the tests. 

The samples’ structure was analyzed by differential scanning calorimeter (DSC) and dynamic mechanical thermal analysis (DMTA). Measurements were carried out with a DSC 204 F1 Phoenix (Netzsch) with a heating rate of 10 °C/min in the temperature range of −100–250 °C in a nitrogen atmosphere. Then, from the second heating, the glass transition temperature (*T_g_*) was determined. The dynamic mechanical thermal analysis (DMTA) was performed using a DMA 242 E/1/G Artemis (Netzsch, Selb, Germany) apparatus working in a bending mode in a temperature range from −100 °C to the polymer melt temperature, at a frequency of 1 Hz and a heating rate of 3 °C/min. The properties were determined based on modulus changes and the ability of attenuation as a function of temperature and frequency of load changes. Since all samples were found to be amorphous, the softening temperatures of the samples were determined using the Boetius apparatus according to the procedure described elsewhere [14,40,41]. 

The thermo-oxidative stability of the polymer blends was evaluated by Thermogravimetric Analysis (TGA 92-16.18 Setaram, Caluire-et-Cuire, France). Measurements were carried out in an oxidizing atmosphere i.e., dry, synthetic air (N_2_: O_2_ = 80: 20 vol. %) at a flow rate of 20 mL/min. The study was in the temperature range of 20–700 °C at the heating rate of 10 °C/min. Additionally, the intrinsic viscosity and mechanical properties of PET-G/PEF blends were measured. The intrinsic viscosity (IV) of the series of blends was determined at 30 °C in the mixture of phenol/1,1,2,2-tetrachloroethane (60/40 by weight). The concentration of the polymer solution was 0.5 g/dl. The measurement was carried out using a capillary Ubbelohde viscometer (type Ic, K = 0.03294).

The static mechanical properties were evaluated using a universal tensile test machine (Autograph AG-X plus, Shimadzu) equipped with an optical extensometer at room temperature, according to EN ISO 527. The stress–strain curves were obtained at a strain rate of 5 mm/min. For each material, five measurements were performed, and then, the results were averaged.

Finally, the water absorption tests were conducted in cold and boiling water, in accordance with the procedures recommended in ASTM D570. The dumbbell shape samples were dried to constant mass at 50 °C within 24 h, cooled to the room temperature, and weighed. To evaluate cold water absorption, the specimens were immersed in distilled water at 23 °C for 24 h. Boiling water immersion was conducted for 30 min, then specimens were allowed to cool down to room temperature in distilled water. All surface water was removed with filter paper and samples were weighed. Each reported value is an average of five test specimens.

## 3. Results and Discussion

### 3.1. Solubility Assessment

According to the well-known concept, the thermodynamic miscibility of two polymers can be estimated by the difference of their solubility parameters [2]. Moreover, polymers, depending on the temperature, may exhibit the phenomenon of phase solubility and phase separation, which is due to the large differences between the phase transition temperatures. The characteristic performance of polymer blends is that in the cooled state, they form a multiphase structure resulting from micro and nanophase separation, characterized by thermodynamic immiscibility [42,43]. This means that two polymers with more similar chemical structures exhibit higher potential that one dissolves in the other. Additionally, what is important, the multiphase polymer blends may exhibit good functional properties if they are compatible/miscible. Several methods allow estimating of the solubility parameters (δ) [4]. 

The Hoy solubility parameters’ method is one of the simplest ways to estimate whether one material is miscible or soluble with/in another organic material (solvent, polymer, etc.,) [4,37]. This method is mostly utilized for structural features like cis, trans (around double bonds), ortho-, meta-, para-substitution (aromatics), and branching (conjugation of double bonds, and rings) [44]. Additionally, the prediction of the solubility parameter is based on three different contributions: a solubility parameter due to dispersion forces (*δ_d_*), all of which are non-polar contributions; a polar contribution (*δ_p_*) due to dipole forces; a hydrogen bond contribution (*δ_h_*), only present when the molecule can form hydrogen bonds or due to donor–acceptor interactions. The Hoy system contains four additive molar functions, several auxiliary equations, and the final expressions for *δ_tot_* and its components *δ_tot_*. Each of these can be regarded as a vector in three-dimensional space, so the total solubility parameter *δ_tot_* is defined by Equation (1) [4]:

(1) $$\delta_{tot}^{2}=\delta_{d}^{2}+\delta_{p}^{2}+\delta_{h}^{2}$$

The full equation that determines the solubility of amorphous polymers (P_i,j_) [4]:

(2) Δδ=[(δd,Pi−δd,Pj)2+(δp,Pi−δp,Pj)2+(δh,Pi−δh,Pj)2]12

where Δδ is a difference in the solubility parameters of polymer pairs, and *δ_d_*, *δ_p,_* and *δ_h_* are resulting from dispersion forces, polar interaction, and hydrogen bonding. The smaller difference in solubility of block pairs Δδ≤5 MPa^1/2^, the more soluble they are [4]. It is possible to calculate the theoretical solubility parameters for polymers compositions.

**Figure 2:**
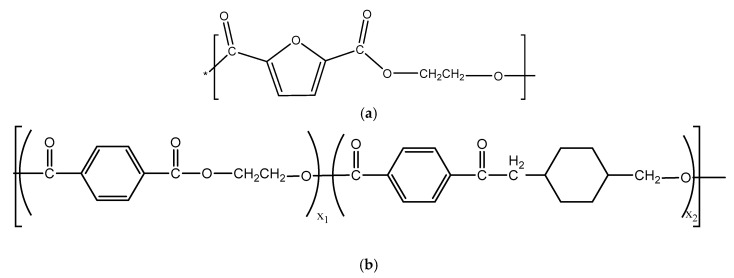
Molecular structures of PEF (**a**) and PET-G (**b**).

The molecular structures of the PEF and PET-G are shown in Figure 2a,b, respectively, and the total solubility parameters and its components were calculated by using Hoy’s method (Table 1). 

According to the calculations followed by Hoy’s method, the prepared polymer blends exhibit higher values of Δδ parameter than the parameter appointed for completely miscible materials. This proves that in the PET-G/PEF system, the homopolymers are immiscible and exhibit phase separation. However, the obtained value of the Δδ parameter is only slightly higher, so one can expect partial miscibility. Based on these calculations, it can be deduced that in the cooled state, the blends form a heterogeneous structure with two separate phases.

### 3.2. Analysis of Interfacial Interactions

**Figure 3:**
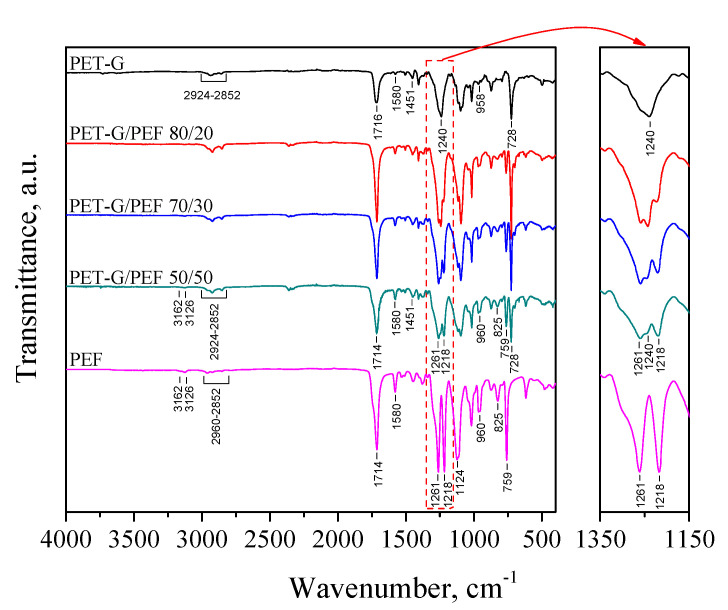
FTIR spectra of neat PET-G, PEF, and PET-G/PEF blends.

To confirm the molecular structure of PET-G/PEF blends, FTIR spectroscopy was carried out. The characteristic FTIR spectra of neat polymers and PET-G/PEF blends are provided in Figure 3. The spectrum of PET-G exhibits significant absorption peaks at 1716 and 1240 cm^−1^, corresponding to =C=O and =C(=O)–O– stretching of ester groups, respectively. Signals arising from methylene groups’ bending mode appear at 1451 cm^−1^, whilst those of symmetrical and asymmetrical stretching of CH_2_ occur in the range of 2924–2952 cm^−1^. Aromatic carbon-carbon stretching vibrations are centered at 1580 cm^−1^, whereas ring C-H out-of-plane bending occurs as a strong band at 728 cm^−1^ [45,46]. Moreover, absorption peaks originating from the C-H stretching mode of the cyclohexane ring can be observed at 958 cm^−1^ [46,47]. All signals characteristic for PET-G are also evident at blend spectra. Besides, at PET-G/PEF spectra, weak absorption peaks appear at 3126 and 3162 cm^−1^ due to =C-H stretching vibrations, typical for the furan ring in PEF [48,49]. Moreover, new signals arising from the =C–O–C= stretching mode of ester groups and =C–O–C= ring vibrations of furan ring occur at 1261 and 1218 cm^−1^, respectively (see the enlarged view of Figure 3). Finally, several bands ascribed to out-of-plane deformation of 2,5-disubstituted furan heterocycle are depicted at 960, 825 cm and 759 cm^−1^ [50]. While comparing blends spectra, one may note that along with an increase in PEF weight fraction, the absorption bands corresponding to =C–O–C= stretching (1261 cm^−1^) and =C–O–C= (1218 cm^−1^) ring vibrations became more intense, resembling more and more PEF homopolymer features. 

### 3.3. Morphology of PET-G/PEF Blends

**Figure 4:**
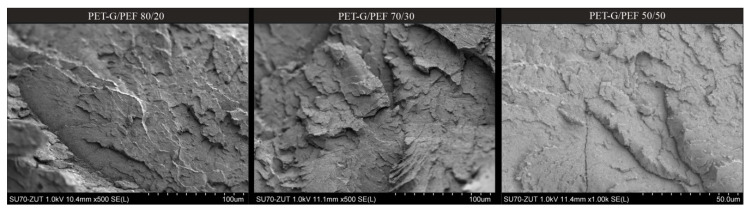
SEM images of PET-G/PEF reactive blends at 80/20; 70/30; 50/50.

The SEM micrographs of fracture surfaces (Figure 4) of the series of PET-G/PEF blends supposed evidence of changes in morphology, as well as provided confirmation of the calculation of miscibility parameters and observations made based on FTIR analysis. SEM micrograms did not explicitly confirm the two-phase structure of the analyzed systems, in which the separation of phases is usually observed, e.g., in the case of PET/PP [51] systems or PET/PBT [52]. All samples were characterized by a comparable, more homogenous morphology—complete homogeneity between PET-G and PEF is thermodynamically unobtainable, due to the differences in chemical structure, molecular mass, density as well as polarity, which was confirmed by the calculations of solubility parameters (Hoy’s method). In our opinion, since both polymers included in the blend exhibit the value of theoretical solubility parameters (Δδ) approximately equal to 5, one can assume that probably PEF can penetrate and dissolve in PET-G and thus, domains are fused and the boundaries between phases are blurred. Observations made based on SEM micrograms suggest that there is strong interphase between PET-G and PEF, with noticeably lower surface tension, interconnecting, and “stitching” of both phases. Therefore, some changes in the structure of the materials, analyzed by DSC and DMTA methods, as well as the impact on the functional properties of blends, such as thermal stability, mechanical properties and water absorption, will be expected.

### 3.4. Structural and Thermal Properties

**Figure 5:**
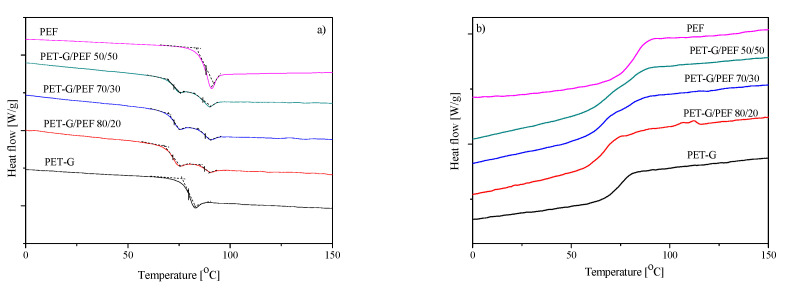
DSC thermograms recorded during 2nd heating (**a**) and cooling (**b**).

DSC and DMTA analyses of polymer blends were performed to evaluate phase transition temperatures, as well as their thermal effects. Changes in the thermal transition temperatures of polymer blends relative to the neat polymers can provide evidence of interaction between the components [53]. Figure 5 depicts the DSC non-isothermal curves for the post-consumer PET-G, PEF, and series of PET-G/PEF blends. Additionally, the values of glass transition temperatures, the corresponding heat capacity, as well as the values of Boetius softening temperatures are summarized in Table 2. The obtained data show that post-consumer PET-G had a glass transition temperature lower by 7 °C than the synthesized PEF. The thermal properties of PEF, i.e., *T_m_* around 210 °C and *T_g_* around 80 °C, have been known for over a decade [54]. Differences in the *T_g_* values can be seen in the molecular structure of both polymers. FDCA is strikingly similar to terephthalic acid, and differences in ring size, polarity, and linearity result in significantly different performance metrics [22]. Besides, the nonlinear character in FDCA combined with the permanent dipole frustrates the crystallization process, resulting in the slow isothermal crystallization rates observed in [55,56]. Therefore, on DSC curves recorded at the heating rate of 10 °C (Figure 5), for both amorphous PET-G and for slowly crystallizing PEF, no melting and crystallization peaks were observed. In turn, the values of *T_g_* for the prepared blends are primarily due to two factors: the mixing of two unmixed polymers with different glass transition temperatures and the processing processes carried out (especially the extrusion process). Therefore, in the obtained blends, the *T_g_* of the PEF component remains practically unchanged (within the limits of measurement error), whilst the reduction by 8 °C (10%) in the *T_g_* value for the PET-G component results precisely from the processing carried out for this post-consumer material. Besides, the corresponding of glass transition to the values of heat capacity showed that both homopolymers exhibited the same value of ΔC_p_ of 0.49 J/g∙°C, while the blends were dependent on the number of components, i.e., along with an increase in PEF, the value of ΔC_p_ increased. These observations are in the agreement with the study of Momanyi et al. [57], that the *T_g_* for recycled PP was ∼12.9% lower than that of virgin PP due to several factors, such as reprocessing conditions, thermal history factors, the presence of less perfect crystals, chain degradation that occurs during melt processing, and other changes in crystals [57]. Moreover, because, under the DSC test conditions, all materials did not show crystallization and melting peaks, to examine at what temperature a given material begins to “flow”, which was useful to determine the conditions of injection molding, softening temperatures were determined using a Boetius apparatus. Therefore, it was also shown that the most thermally stable is PEF, with *T_B_* equal to about 197 °C. The post-consumer PET-G had two characteristic temperatures: the onset of softening point (124 °C) and the temperature when the sample had practically run off the microscope slide (140 °C). The prepared blends showed only one temperature, softening temperature, and its value increased with the increase in the PEF content in the blend. 

**Figure 6:**
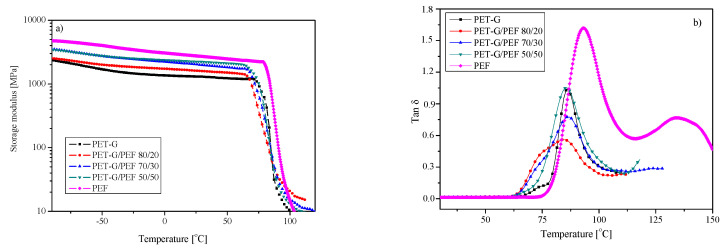
The storage modulus E` (**a**) and tan δ (**b**) curves a function of temperature for PET-G/PEF blends.

The results of DMTA for the series of PET-G/PEF blends are presented in Figure 6, where the curves of storage modulus (*E`*) as the function of temperature (T) at 1 Hz are presented in Figure 6a, while the tan δ curves as the function of temperature are presented in Figure 6b. These observations are in agreement not only with the data obtained from DSC, but also with the study of Codou et al. [58], who compared the glass transition dynamics and cooperativity length of PEF and PET. The DMTA curves for the prepared samples (homopolymers and blends) present three major thermomechanical phenomena (Figure 6). First, a significant decrease in E` has been noticed in the temperature range of 62–107 °C, which is attributed to the α-relaxation process (Figure 6a). This drop is higher for PEF than for PET-G and PET-G/PEF blends, which is consistent with previous observations [22,58] and is likely caused by the lower chain entanglement density in PEF. The second phenomenon highlighted only on the curve of PEF and slightly for PET-G/PEF 50/50 (Figure 6b) results from an increase in the modulus that can be attributed to cold crystallization on heating. Finally, the last drop of modulus is associated with the melting of crystals in the case of PEF. Moreover, the value of E` in 25 °C testifies to a much greater PEF stiffness than PET-G (Table 2). It is visible that along with an increase in PEF content in the blends, the values E` in 25 °C also increase. What is surprising from the tan *δ* curves (Figure 6b) is that the blends of two immiscible polymers exhibited one peak corresponding to one glass transition. It is well known that the *T_g_* of a polymer in immiscible blends does not change with composition and is expected to maintain its bulk value, whereas in miscible blends, the *T_g_*’s of the components move towards each other [59]. A similar phenomenon has already been observed in the works of Tirtha et al. [60], where the *T_g_* of polystyrene in a blend with both PP as well as PE was shown to change with composition, and consequently, the morphology, even though these blends were known to be immiscible. In addition, the values of *T*_α_, temperature of α-relaxation corresponding to the glass transition determined from tan *δ* curve (Table 2) presents that all blends exhibited one glass transition temperature and this value was comparable to PET-G. Probably, for these two polymers, which are immiscible and are not supposed to react with each other chemically, may occur entanglements of component chains at the interface due to the high processing temperatures (i.e., temperature of extrusion of 170 °C and temperature during injection molding, since the dumbbell shape samples were used in the case of DMTA analysis). These physical interactions at the interface were apparently enough to entangle PEF and PET-G chains and prevent the chains from reorganizing, which was probably also observed in the SEM micrographs (Figure 4).

**Figure 7:**
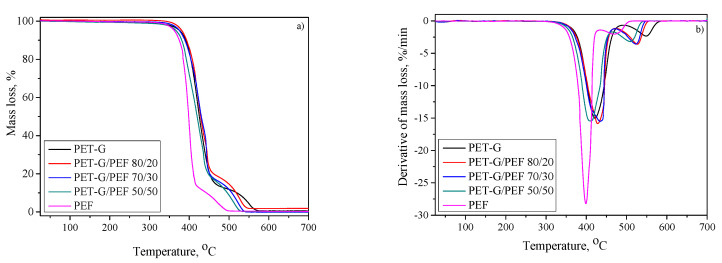
Mass loss (**a**) and derivative of mass loss (**b**) for the series of PET-G/PEF blends.

Moreover, one can apply TGA to analyze the thermal decomposition of polymer blends and this can be related to the thermal stability of the neat polymers. The thermal stability and especially the thermo-oxidative stability of polymer materials is an important factor that could limit their future application. Therefore, in the present study, the thermal behavior of PET-G, PEF, and PET-G/PEF blends was investigated in an oxidizing atmosphere. The mass loss and the first derivative of mass loss curves are shown in Figure 7a,b, respectively. A two-step decomposition profile can be observed in both polyesters and their blends. The first stage located in the temperature range of 295–465 °C originates from the decomposition of polymers’ backbone i.e., chain scission of the ester bonds, whilst the second step at a temperature higher than 470 °C is due to the oxidative decomposition of residue [60,61,62]. The fact that the major decomposition of macromolecular chains of both polyesters merges into one step is consistent with SEM and DMTA observations and may be related to the entanglements of polymer chains at the interface.

The initial decomposition temperature (T_d,5%_) and the temperatures of maximum decomposition rates, obtained from the first derivative of the TGA curve (T_d,DTG1_ and T_d_,_DTG2_), are given in Table 2. From the obtained results, it appeared that PEF is more sensitive to thermal degradation than PET-G. In particular, PET-G exhibits T_d,5%_ of approximately 376 °C, whilst PEF starts to degrade at 361 °C. Therefore, one may expect that the blending of PET-G with furanic polyester will result in a decrease in the thermal stability in blend systems. Intriguingly, from the collected results, it is apparent that the introduction of PEF at lower concentrations has the opposite effect, namely it is beneficial for thermal stability. In particular, the onset decomposition temperature increased from 376 to 381 °C in the PET-G/PEF 80/20, while in the blend containing 20% PEF, T_d,5%_ is nearly comparable to the temperature characteristic for the PET-G homopolymer. Moreover, this improvement is also reflected in the values of decomposition temperatures at the maximum rate (T_d,DTG1_). The T_d,DTG1_ of the 80/20, and 70/30 wt.% PET-G/PEF blends were delayed by 8 and 13 °C, respectively, when compared to that of recycled PET-G (421 °C). This might be due to potential interactions between polymers at a specific concentration (80/20 and 70/30). PET-G chains could form a network structure with the PEF by reacting with their functional groups (-COOH, -OH) which are the precursors of the degradation. Therefore, the degradation process is hindered and the overall stability of the blend is improved [63]. A similar synergistic effect in the improvement of thermal stability on polymer blends was previously observed among others by Calderon and Sobkowicz [53] in poly(propylene carbonate)/polyoxymethylene blends, and by Ciro et al. [64] in recycled rubber/recycled polypropylene blends. Further increase in the PEF content up to 50% depresses the temperatures of both T_d,5%,_ and T_DTG1_ towards values lying in-between temperatures found for homopolymers. As far as decomposition of residue is concerned, it has been found that temperatures corresponding to the maximum rate of mass loss (T_d, DTG2_) are exhibiting much more pronounced behavior, shifting systematically to lower temperature along with the increasing fraction of PEF. Moreover, at this point, it is worth emphasizing that almost all investigated samples decompose completely at a temperature close to 675 °C (0% char residue). The PET-G/PEF 80/20 is the only exception, in which residual mass at 700 °C turns out to be higher (~1.9%), suggesting the presence of inorganic fraction in this sample. This fact can be rationalized by considering that in this contribution, we are dealing with recycled material, of which preparation for processing is quite complex, and its final properties depend on the purity of the stock. Most likely, this small fraction of inorganic matter may be the result of incomplete removal of printing ink, rich in inorganic fillers such as talc or SiO_2_ [65]. What is more, the latter may also contribute to an increase in the polymer thermal stability [66,67]. 

That is to say, the TGA analysis does not allow us to make a simple and clear conclusion about the influence of blend composition on thermal properties, since some additional issues have to be taken into account when recycled materials are considered. Nevertheless, the onset of decomposition temperatures is well above the softening temperatures (T_B_) of investigated blends, ensuring their safe processing by conventional techniques, such as extrusion or injection molding. 

### 3.5. Mechanical Properties

**Figure 8:**
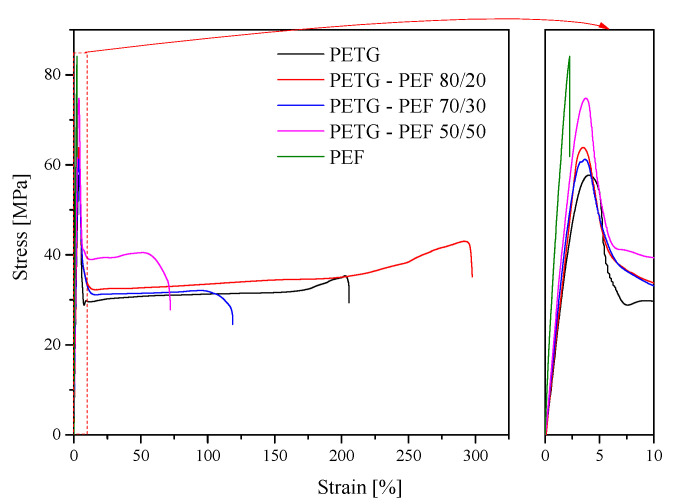
Representative stress–strain curves for the series of PET-G/PEF blends.

The representative stress–strain curves for both neat polymers and PET-G/PEF blends are presented in Figure 8. Besides, Table 3 summarizes the results of stress–strain data, i.e., Young’s modulus (E, calculated from strain 0.05% to 0.25%), tensile strength and elongation at yield (σ_y_ and ε_y_, respectively), strength and elongation at break (σ_b_ and ε_b_, respectively), as well as the values of intrinsic viscosity (IV). Besides, following Brostow et al. [68,69], who demonstrated the existence of a quantitative relationship between toughness (τ) and the brittleness (B), valid for polymers exhibited a wide range of chemical structures and properties, one calculated B accordingly to Equation (3):

(3) $$B=\frac{1}{\varepsilon_{b}\cdot E`}$$

where *ε_b_* is the elongation at break and *E`* is the storage modulus determined by DMTA at 1 Hz and 25 °C; the values of *B* are also summarized in Table 3.

It was revealed that the values of IV for PET-G and PEF were 0.590 and 0.503, respectively. In turn, for the series of blends the values of IV resulted from the number of components, i.e., along with an increase in the amount of PEF, the value of IV decreased. The value of IV for post-consumer PET-G of 0.59 was comparable to the one obtained by Zhang et al. [70], i.e., the recycled PET-G (Shengxin Co., China), prepared from waste films and liquid bottles through several procedures such as breaking up, washing, heating, etc., had an intrinsic viscosity of 0.58 dL/g, while the original PET-G usually had an intrinsic viscosity of approximately 0.7 dL/g. The synthesized PET-G, in our laboratory in the 1 dm^3^ steel reactor, following the 2-staged polycondensation process, had an IV of 0.696 dL/g [41,46]. As expected from the IV values, the newly synthesized PET-G exhibited higher IV than the post-consumer PET-G, which had been subjected to heat and shear during the recycling procedure. In turn, the neat PEF had a slightly lower value of IV that the post-consumer PET-G. However, the PEF synthesized by polycondensation in the melt, exhibited a significantly higher value of IV in comparison to PEF synthesized by solid-state polymerization (SSP), wherein depending on the used catalyst, different intrinsic viscosity of 0.30, 0.31, and 0.38 dL/g were measured [71]. Meanwhile, Hong et al. [72] using a two-step melt polycondensation procedure similar to us, but higher temperatures of the second stage and a different catalyst, obtained the value of IV of 0.60. This confirms that both the choice of polymer preparation method and, above all, the reaction conditions, including the choice of catalyst, clearly affect IV values. Nevertheless, as already mentioned, in the case of PET-G/PEF blends, the incorporation of an increasing amount of PEF (lower value of IV) in PET-G causes a decrease in the IV value, which results from the law of mixtures.

In turn, PEF exhibited over three times higher value of E in comparison to PET-G, while in the prepared blends, as expected, along with an increase in the amount of PEF, an increase in E was observed. Similarly, the values of tensile strength at yield and strength at break also depend on the amount of PEF. However, for the homopolymer PEF, one could not observe the tensile strength and elongation at yield. Moreover, as expected based on DSC and DMTA results, PEF was the most rigid, and this was confirmed by the value of elongation at break. This is in the agreement with the study of Burgess et al. [22], who confirmed that a bio-sourced polyester derived from 2,5-furandicarboxylic acid (PEF), exhibited improved mechanical properties, a higher glass transition temperature, and slower chain mobility than its terephthalic acid counterpart. Besides, herein PEF at the same time exhibited the highest value of strength at break and the lowest value of elongation at break if compared to neat PET-G and PET-G/PEF blends. In the case of PET-G/PEF blends, the values of elongation at yield were comparable to each other and comparable to PET-G, whilst the values of elongation at break initially increased to 297% (PET-G/PEF 80/20), perhaps due to occurrence of entanglements of component chains at the interface, and then, gradually decreased as the content of more rigid polyester, namely PEF, increased. 

Besides, the brittleness of the prepared PET-G/PEF blends has been evaluated, since this descriptor turned out to be useful in evaluating polymer blends [73], multi-layer laminate composites [74] and many more [69]. Herein, one can observe (Table 3) that the values of B are in good agreement with the tensile properties, i.e., the highest value of modulus (5.43 MPa) and at the same time, the lowest value of elongation at break (~2%) was observed for PEF. Since the value of E for PET-G and the blends are comparable to one another, only PEF exhibited almost two times E value and significantly higher stiffness (strength at break). Thus, one concludes, that the blends of PET-G/PEF, and especially the system of 50/50 exhibits the most promising properties, taking into account both high modulus, elongation at break, and brittleness.

### 3.6. Water Absorption 

**Figure 9:**
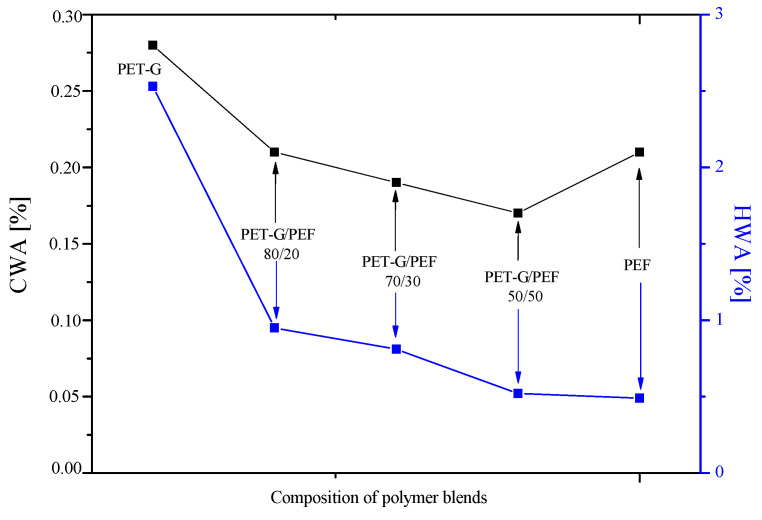
Cold and hot water absorption measurements for the series of PET-G/PEF blends.

The absorption of water and moisture by polymer materials is governed by several different mechanisms [75,76]: (1) the diffusion of water molecules inside the microgaps or free volume between polymer chains; (2) the capillary transport of water molecules into space between two phases due to the imperfect interfacial bonding between the phases, especially when they are of different polarities and immiscible with each other; (3) and recently, solubility, especially in amorphous polymers of low to moderate hydrophilicity [76]. The results of cold water absorption (CWA) and hot water absorption (HWA) are shown in Figure 9. For each sample, three measurements were performed, and the measurement error is ca. 0.002. It is visible that PEF exhibits a lower value of CWA of about 30% in comparison to PET-G. In the case of PET-G/PEF blends, along with an increase in PEF content, the CWA decreases. The lowest value of CWA was obtained for PET-G/PEF 50/50, and it was lower in comparison to both the neat PET-G and neat PEF. In turn, PEF indicates more than five times the lower value of HWA compared to PET-G. More importantly, PET-G/PEF blends show comparable to PEF boiling water absorption values. It was shown that in PET-G/PEF blends, along with the increase in PEF content, the absorption of boiling water significantly decreased by 60 to 80%, for the blends containing 20 to 50 wt. % PEF, respectively. Burgess et al. [23] proved that PEF exhibited a significantly reduced water diffusion coefficient of ~5 times compared to PET at 35 °C. The reduction in diffusion coefficient for PEF in comparison to PET originated from the reduction in segmental mobility due to the non-symmetrical furan ring in PEF compared to the symmetrical phenyl ring in PET [22] or PET-G [41]. Nevertheless, significant improvement in water absorption in PET-G/PEF blends can be due to the fact that capillary transport of water molecules into space between PET-G and PEF was limited by the existence of interfacial interactions between phases. Moreover, even though both phases were of different polarities and were immiscible with each other, one could have observed the significant improvement of barrier properties toward the water. These observations may confirm that the blends of post-consumer PET-G foils with bio-based PEF, especially the PET-G/PEF 50/50, might be an alternative to PET used in the beverage contained market and allow the utilization of PET-G foils, and thereby, reduce the waste stream entering landfills.

## 4. Conclusions

This work was devoted to the analysis of the novel, environmentally friendly melt compounded PET-G/PEF blends with varied component weight ratios. Polymer blending enables the recycling of polymers, thus, preventing the use of natural resources and reducing environmental pollution. PET-G is one of the most commonly used thermoplastic polyesters for the mass production of packaging, disposable dishes, perishables, etc. In turn, PEF as an attractive, viable bio-sourced substitute for PET, is now dominating the packaging market, mainly in the soft drink bottle area due to its superior oxygen and carbon dioxide permeability. Therefore, polymer blends of post-consumer PET-G foil and bio-based PEF, with the comparable values of glass transition temperatures, are of special interest not only for the scientific community but also for industry. Despite the fact that the calculations made according to Hoy’s method appointed for completely immiscible materials, the SEM and FTIR analyses, supported by DSC and DMTA results, indicated the potential for the specific entanglements between the polymers due to the high processing temperature, which was reflected in a positive hybrid effect on the thermal stability and reduced water diffusion of the blends. Moreover, it was found that both phase transition temperatures and mechanical parameters depended on the composition of the blend. Obtaining this type of material is an extremely important issue for managing environmentally troublesome polyester foils by mixing them with bio-based thermoplastic polyester. Considering both the synergistic effect of improving thermal properties for the PET-G/PEF 80/20 and the water diffusion for the PET-G/PEF 50/50, with the optimum characteristic phase transition temperatures and mechanical parameters for the blends 80/20 and 70/30, and above all, the market price of dimethyl furan-2,5-carboxylate of about USD 800/kg [77], the most promising system is the one containing 80 wt. % PET-G and 20 wt. % PEF. A circular economy for plastic materials, especially PET’s derivatives, like PET-G or PEF, is one that would ‘close the loop’ by recycling used foils or bottles, then using that recovered plastic to form new products that are not necessarily bottles or foils. Taking into account all functional properties (mechanical and water absorption) along with the price of FDADM, the blend PET-G/PEF 80/20 can be successfully applied for foils and packaging containers (e.g., packaging of new phones and chargers), garden tunnels (plastic greenhouses for growing plants), polyester weed control fabrics, filament for 3D printing, polyester yarns and fibers (e.g., textiles, transporting bags, industrial “big-bags” etc.,) and composites, as both reinforcing phase and matrix.

## Figures and Tables

**Table 1:** Solubility parameters of the PET-G/PEF blends calculated by using Hoy’s method.

Solubility Parameters	PET-G [MPa^1/2^]	PEF [MPa^1/2^]
δ_tot._	33.87	31.30
δ_p_	13.88	16.95
δ_h_	23.38	20.75
δ_d_	20.20	16.19
Δδ_PET-G/PEF_		5.70

**Table 2:** Structural and thermal properties of PET-G/PEF reactive blends.

Sample	T_g_ [°C]	ΔCp [J/g∙°C]	T_B_ [°C]	E’ at 25 °C [MPa]	Tα (tan δ) [°C]	T_d,5%_ [°C]	T_d,DTG1_ [°C]	T_d,DTG2_ [°C]
PET-G	80	0.49	T_B1_=124 ± 2 T_B2_=140 ± 3	1307	86	376	421	549
PET-G/PEF 80/20	T_g1_=72 T_g2_=87	ΔCp_1_=0.29 ΔCp_2_=0.14	127 ± 3	1607	85	381	429	528
PET-G/PEF 70/30	T_g1_=72 T_g2_=87	ΔCp_1_=0.24 ΔCp_2_=0.18	137 ± 3	2020	86	375	434	522
PET-G/PEF 50/50	T_g1_=72 T_g2_=86	ΔCp_1_=0.17 ΔCp_2_=0.23	174 ± 4	2247	86	367	410	509
PEF	87	0.49	197 ± 4	2755	93	362	399	470

T_g_—glass transition temperature and the corresponding heat capacity ΔCp; T_B_—softening temperature according to Boethius method, where T_B1_—the edges of PET-G sample blur and T_B2_—PET-G starts to soften; E’—storage modulus at 25 °C and 1 Hz; Tα—a temperature of α-relaxation corresponding to the glass transition determined from tan δ curve; T_d,5%_—temperature of the onset of decomposition corresponding to 5 wt. % weight loss; T_d,DTG1_, T_d,DTG2_—the temperature of the maximum rate of weight loss for the first and second decomposition step, respectively.

**Table 3:** Intrinsic viscosity (IV), tensile properties, and the brittleness (B) of the series of PET-G/PEF blends.

Sample	IV [dL/g]	E [MPa]	σ_y_ [MPa]	ε_y_ [%]	σ_B_ [MPa]	ε_B_ [%]	B (10^12^∙(%∙Pa))
PET-G	0.590	1.47 ± 0.02	57.69 ± 0.28	3.96 ± 0.23	35.32 ± 0.43	205.95 ± 2.87	3.7150
PET-G/PEF 80/20	0.590	1.84 ± 0.14	63.83 ± 2.09	3.50 ± 0.09	43.01 ± 0.92	297.55 ± 30.18	2.0913
PET-G/PEF 70/30	0.582	1.98 ± 0.08	60.67 ± 0.65	3.27 ± 0.24	32.36 ± 2.37	115.17 ± 5.43	4.2984
PET-G/PEF 50/50	0.558	2.47 ± 0.03	74.78 ± 0.30	3.75 ± 0.19	37.83 ± 1.37	82.98 ± 7.47	5.3632
PEF	0.503	5.43 ± 0.03	-	-	84.07 ± 4.43	2.27 ± 0.14	159.9015

IV—intrinsic viscosity; E—Young’s Modulus (calculated from strain 0.05% to 0.25%); σ_y_, ε_y_—tensile strength and elongation at yield; σ_b_, ε_b_—strength and elongation at break, respectively; B—brittleness.
